# Assessment of Guideline-Nonconcordant Radiotherapy in Medicare Beneficiaries With Metastatic Cancer Near the End of Life, 2015-2017

**DOI:** 10.1001/jamahealthforum.2021.4468

**Published:** 2022-01-14

**Authors:** Patricia Mae G. Santos, Noah J. Mathis, Kaitlyn Lapen, Stephanie Lobaugh, Divya Yerramilli, Justin E. Bekelman, Erin F. Gillespie

**Affiliations:** 1 Department of Radiation Oncology, Memorial Sloan Kettering Cancer Center, New York, New York; 2 Department of Epidemiology and Biostatistics, Memorial Sloan Kettering Cancer Center, New York, New York; 3 Department of Radiation Oncology, Abramson Cancer Center, Hospital of the University of Pennsylvania, Philadelphia; 4 Center for Health Policy and Outcomes, Memorial Sloan Kettering Cancer Center, New York, New York

## Abstract

This cross-sectional study uses data from the Centers for Medicare & Medicaid Services to assess the use of professional society guideline-nonconcordant radiotherapy in patients at the end of life.

## Introduction

Choosing Wisely and professional society guidelines from 2014 to 2017 recommend the avoidance of extended radiotherapy for patients with metastatic cancer.^1,2,3^ This recommendation is perhaps most salient for patients at the end of life, for whom minimizing time on treatment is essential to preserving quality of life.

Despite guidelines supporting shorter regimens for metastatic disease, adherence has been limited.^4^ Prior research^4^ focused on all patients regardless of prognosis. We assessed the use of guideline-nonconcordant radiotherapy in patients at the end of life. We hypothesized that although guidelines specify avoidance of extended radiotherapy for metastatic disease, such regimens may still be used at or near the end of life.

## Methods

In this cross-sectional study, we assessed a limited data set of radiotherapy episodes released by the Centers for Medicare & Medicaid Services in July 2019 for the Radiation Oncology Alternative Payment Model. We limited our analysis to data from patients aged 65 years or older who died within 90 days of treatment planning. Data on race and ethnicity were not available in the data set used for this study. An exemption was obtained from the Memorial Sloan Kettering Cancer Center institutional review board for analysis of this data set. Data were analyzed in April 2021. This study followed the Strengthening the Reporting of Observational Studies in Epidemiology (STROBE) reporting guideline for cross-sectional studies.

The primary outcome was guideline-nonconcordant radiotherapy, defined as a greater than 10-fraction regimen or combination regimen (including conventional external beam radiotherapy in combination with stereotactic radiosurgery for brain metastases).^1,2,5^ The eFigure and eMethods in the Supplement present details of the data set, consort diagram, and analysis plan. In brief, a logistic regression adjusting for select variables was used to identify factors associated with guideline-nonconcordant regimens. A *P* value less than .01 indicated statistical significance owing to multiple comparisons. Data were analyzed with R software version 3.6.1 (R Foundation for Statistical Computing).

## Results

Bone and brain metastases comprised 16% of radiotherapy episodes (70 153 of 467 781 episodes). Of 517 988 total radiotherapy episodes in the database, our analysis was limited to 46 781 episodes among patients aged 65 years and older (43 196 with bone metastases [9.2%] and 26 957 with brain metastases [5.8%]). Of these, 17 482 episodes (3.7%) were associated with patient death within 90 days of radiotherapy (bone metastases, 8966 of 17 482 [51.3%]; brain metastases, 8516 of 17 482 [48.7%]). Of 17 482 radiotherapy episodes, 9839 were in male patients (56.0%) and 7613 were in female patients (44.0%); 9192 were in the group aged 65 to 74 years. Of those patients who died within 90 days of receipt of radiotherapy, 13 688 patients (78.4%) received guideline-concordant radiotherapy (7118 with bone metastases [52.0%] and 6570 with brain metastases [48.0%]), and 3764 (21.6%) received guideline-nonconcordant radiotherapy (1829 with bone metastases [49.0%] and 1935 with brain metastases [51.0%]).

Unadjusted proportions of guideline-nonconcordant radiotherapy are depicted in the Figure. In the multivariate analysis, factors associated with reduced adjusted odds ratios (aORs) of guideline-nonconcordant radiotherapy included treatment in hospital-affiliated facilities (aOR, 0.50; 95% CI, 0.44-0.56; *P* < .001) and older patient age (age 75-85 years vs 65-75 years: aOR, 0.90; 95% CI, 0.83-0.97; *P* < .001; age ≥85 years vs 65-75 years: aOR, 0.73; 95% CI, 0.64-0.84; *P* < .001) (Table). Factors independently associated with greater aORs of guideline-nonconcordant radiotherapy included receipt of major procedures (aOR, 1.17; 95% CI, 1.05-1.32; *P* = .01), chemotherapy (aOR, 1.26; 95% CI, 1.12-1.41; *P* < .001), and survival more than 30 days after the treatment planning appointment (aOR, 4.72; 95% CI, 4.11-5.44 for 31-60 days compared with 1-30 days, aOR, 6.55; 95% CI, 5.70-7.56 for 61-90 days compared with 1-30 days; *P* < .001).

**Figure.**
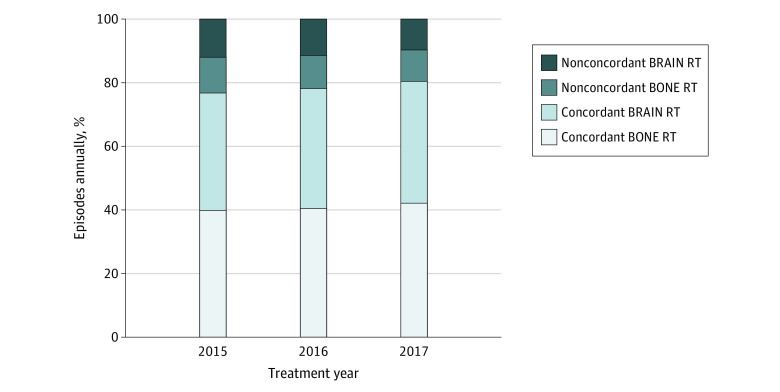
Guideline Concordance in Radiotherapy for Metastatic Cancer Near the End of Life, 2015-2017 Abbreviations: BONE RT, bone metastases radiotherapy; BRAIN RT, brain metastases radiotherapy.

**Table.** Probability of Guideline-Nonconcordant Radiotherapy

Variable	No. (%)			aOR (95% CI)	*P* value
	All episodes (N = 17 452)	Concordant (n = 13 688)	Nonconcordant (n = 3764)		
**Patient characteristic**					
Sex					
Female	7613 (44)	5958 (44)	1655 (44)	1 [Reference]	.40
Male	9839 (56)	7730 (56)	2109 (56)	1.03 (0.96-1.11)	
Age group, y					
65-74	9192 (53)	7070 (52)	2122 (56)	1 [Reference]	<.001
75-84	6934 (37)	5065 (37)	1329 (35)	0.90 (0.83-0.97)	
≥85	1866 (11)	1553 (11)	313 (8.3)	0.73 (0.64-0.84)	
**Disease characteristic**					
Metastases					
Bone	8947 (51)	7118 (52)	1829 (49)	1 [Reference]	<.001
Brain	8505 (49)	6570 (48)	1935 (51)	1.60 (1.361.88)	
**Treatment characteristic**					
Major procedure					
No	6099 (35)	4794 (35)	1305 (35)	1 [Reference]	.01
Yes	11 353 (65)	8894 (65)	2369 (65)	1.17 (1.051.32)	
Chemotherapy					
No	9676 (55)	7374 (54)	2302 (61)	1 [Reference]	<.001
Yes	7776 (45)	6314 (46)	1462 (39)	1.26 (1.121.41)	
Year					
2015	5978 (34)	4591 (34)	1387 (37)	1 [Reference]	.02
2016	5855 (34)	4578 (33)	1277 (34)	0.95 (0.811.11)	
2017	5619 (32)	4519 (33)	1100 (29)	0.80 (0.68-0.95)	
Treatment setting					
Freestanding center	6981 (40)	4997 (37)	1984 (53)	1 [Reference]	<.001
Hospital outpatient department	10471 (60)	8691 (63)	1780 (47)	0.50 (0.44-0.56)	
**Clinical outcome**					
Death during the 90-d episode					
In 1-30 d	4010 (23)	3758 (27)	252 (7)	1 [Reference]	<.001
In 31-60 d	7409 (42)	5676 (41)	1733 (46)	4.72 (4.11-5.44)	
In 61-90 d	6033 (35)	4254 (31)	1779 (47)	6.55 (5.70-7.56)	

Abbreviation: aOR, adjusted odds ratio.

## Discussion

In this nationally comprehensive database study of radiotherapy episodes for bone and brain metastases, 22% of episodes delivered within 90 days of death were extended regimens despite guidelines advising against their use. A prior study^6^ suggested that radiation oncologists may wish to avoid shorter courses of radiotherapy for bone metastases owing to concerns regarding the increased risk of retreatment. Longer regimens may be reserved for patients with longer expected survival rates, who may possibly benefit from the durable local control. Patients at the end of life may be selected for shorter treatments. However, results of this study suggest that more than one-fifth of patients with metastatic disease at the end of life may receive guideline-nonconcordant radiotherapy.

Limitations of this study include the use of Medicare claims data without linkage to medical records or cancer registries, so clinical information such as performance status and histologic factors were unavailable. Further, use of combination regimens may reflect treatment of multiple body sites; however, for brain metastases, this combination most likely reflects stereotactic radiosurgery with adjuvant whole-brain radiotherapy, a practice specifically discouraged by Choosing Wisely guidelines.^3^

## Conclusions

Our findings suggest that the burden of unnecessary radiotherapy in the metastatic setting is shared by a sizeable proportion of patients near the end of life, underscoring the importance of guideline adherence. Future research to evaluate interventions to improve guideline concordance for patients with bone or brain metastases in the last 3 months of life, including the effects of the upcoming change to bundled payments under the Radiation Oncology Alternative Payment Model, is warranted.

## Notes

Supplement. **eFigure.** Definition of Study Cohorts and Reasons for Exclusion **eMethods.** Data Analysis
