# Supplementary material for: Assessment of Guideline-Nonconcordant Radiotherapy in Medicare Beneficiaries With Metastatic Cancer Near the End of Life, 2015-2017
Source: JAMA Health Forum. 2022 Jan 14;3(1):e214468. doi: 10.1001/jamahealthforum.2021.4468 (PMC8903107; doi:10.1001/jamahealthforum.2021.4468)
Supplement: Supplement. — eFigure. Definition of Study Cohorts and Reasons for Exclusion eMethods. Data Analysis [file jamahealthforum-e214468-s001.pdf]

Supplemental Online Content

Santos PMG, Mathis NJ, Lapen K, et al. Assessment of guideline-nonconcordant radiotherapy in Medicare beneficiaries with metastatic cancer near the end of life, 2015-2017. *JAMA Health Forum*. 2022;3(1):e214468. doi:10.1001/jamahealthforum.2021.4468

eFigure. Definition of Study Cohorts and Reasons for Exclusion
eMethods. Data Analysis

This supplemental material has been provided by the authors to give readers additional information about their work.

© 2022 Santos PMG et al. *JAMA Health Forum*.

eFigure. Definition of Study Cohorts and Reasons for Exclusion

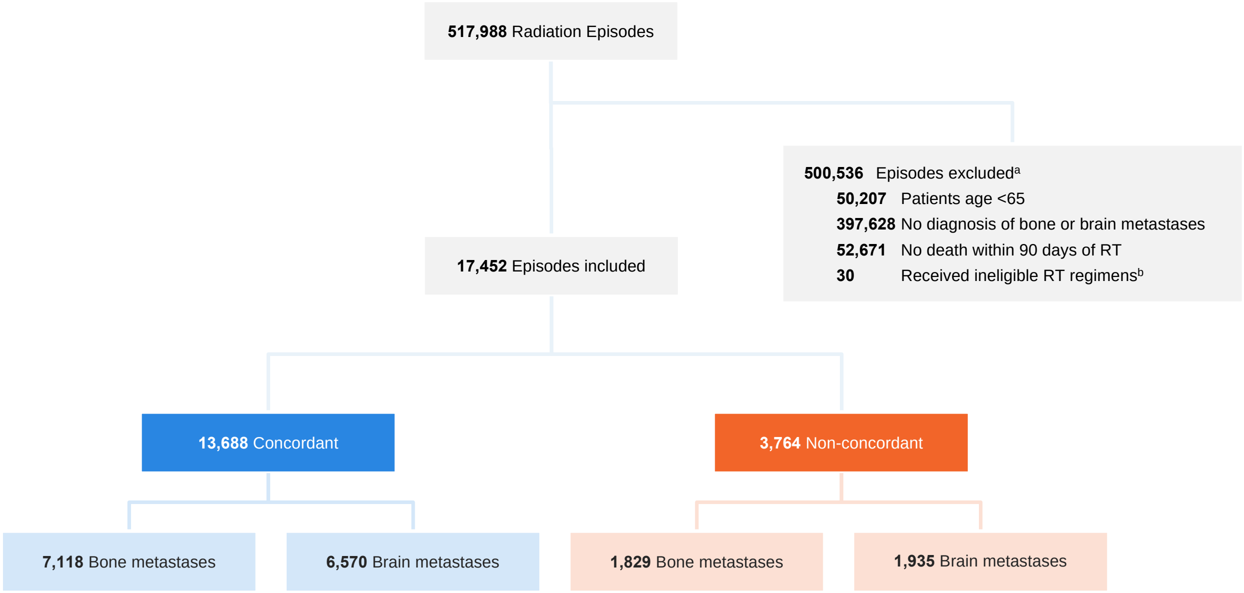

[a] Inclusion criteria were Medicare beneficiaries age ≥65 who received radiation for bone or brain metastases. Exclusions listed were applied sequentially in order of appearance. Patients could be excluded for more than one reason.

[b] Brachytherapy, IORT, >10-fractions of SRS, and PBT comprised <1% of bone and brain metastases episodes and were thus excluded from analysis

© 2022 Santos PMG et al. *JAMA Health Forum*.

eMethods. Data Analysis

The CMS dataset used for our analysis included 517,988 episodes of radiation. This is made up of all radiation episodes for Medicare patients between 2015 and 2017 for 17 cancer diagnoses, representing 84% of all Medicare radiation treatments. The diagnoses included were identified using ICD-9 or ICD-10 codes, and data was presented from insurance claims. Episodes were defined as the 90 days that followed the initial RT treatment planning session, and included all services delivered during that time. If a patient had additional radiation outside of this 90-day window, this was considered a separate episode.

Our analysis included episodes delivered to patients who died within 90 days of RT. Modalities included were EBRT, intensity-modulated radiotherapy (IMRT), stereotactic body radiosurgery (SRS) or radiotherapy (SBRT), and combined radiation regimens, defined as those in which patients received SRS combined with EBRT or IMRT. Brachytherapy, intraoperative radiotherapy (IORT), and proton beam therapy (PBT) comprised <1% of episodes and were excluded. We also excluded patients under the age of 65.

Covariables included in the logistic regression model were diagnosis, gender, age group (grouped into 65-74, 75-84, 85+), diagnosis (bone or brain metastases), receipt of major procedure or chemotherapy within the past 90 days, year of treatment, treatment setting (hospital-affiliated outpatient department (OPD) versus freestanding clinic (FREE)), time from treatment to death (1-30 days, 31-60 days, 61-90 days), and interaction terms between year treated and diagnosis, diagnosis and recent procedure, diagnosis and recent chemotherapy, and treatment setting and year.

© 2022 Santos PMG et al. *JAMA Health Forum.*
